# Glucocorticoid Therapy in Inflammatory Bowel Disease: Mechanisms and Clinical Practice

**DOI:** 10.3389/fimmu.2021.691480

**Published:** 2021-06-03

**Authors:** Stefano Bruscoli, Marta Febo, Carlo Riccardi, Graziella Migliorati

**Affiliations:** Section of Pharmacology, Department of Medicine and Surgery, University of Perugia, Perugia, Italy

**Keywords:** glucocorticoids, IBD, GILZ, inflammation, drug delivery

## Abstract

Inflammatory bowel disease (IBD) comprises ulcerative colitis (UC) and Crohn’s disease (CD). IBD etiopathology is multifactorial and involves alteration of immune cells and chronic activation of the inflammatory cascade against yet unknown environmental factors that trigger the disease. IBD therapy aims at improving the quality of life and reducing the risk of disease-related complications to avoid the need for surgery. There is no specific cure for IBDs, and the focus of therapy is supportive measures and use of anti-inflammatory and immunosuppressive drugs. Glucocorticoids (GCs) are powerful anti-inflammatory and immunomodulatory agents used to treat many acute and chronic inflammatory diseases. GCs remain basic treatment for moderate-to-severe IBD, but their use is limited by several important adverse drug effects. Topical administration of a second-generation of GCs, such as budesonide and beclomethasone dipropionate (BDP), represents a valid alternative to use of older, systemic GCs. Administration of second-generation GCs shows promisingly high topical activity and less systemic toxicity, but maintenance therapy with these new GCs in IBD patients is associated with multiple adverse effects. In this review, we make a comparative analysis of the efficacy of first-generation and second-generation GCs in IBD treatment. Unraveling GC biology at the molecular level to uncouple their clinical benefits from detrimental effects is important. One approach is to consider new GC mediators, such as glucocorticoid-induced leucine zipper, which may have similar anti-inflammatory properties, but avoids the side effects of GCs. This in-depth analysis can help to improve the development and the clinical outcomes of GC therapies in IBD.

## Introduction

Inflammatory bowel disease (IBD) comprises Crohn’s disease (CD) and ulcerative colitis (UC). These are chronic and progressive diseases affecting the gastrointestinal tract. They arise as a consequence of a complex multifactorial etiopathogenesis that is incompletely understood, but which includes genetic predisposition and various environmental factors (1, 2). Oral systemic corticosteroids (e.g., prednisone, prednisolone) have been used to induce remission in IBD patients for more than 60 years due to their powerful anti‐inflammatory effects. Second generation glucocorticoids (GCs), such as budesonide and beclomethasone dipropionate (BDP), which are characterized by high anti‐inflammatory activity, with low systemic bioavailability due to a significant first-pass effect and higher affinity for the glucocorticoid receptor (GR) compared to first generation GCs, represent an important alternative approach for IBD therapy (3). The possible advantage derives mainly from the lower adverse effects following treatment with these second-generation GCs. Nevertheless, the increase in IBD incidence and lack of long-term treatment alternatives have led to a search for new strategies and approaches.

In this review, we discuss the medical uses of synthetic GCs (e.g., second-generation GCs). We also report new perspectives in the study of GCs, focusing especially on the role of the glucocorticoid-induced leucine zipper (GILZ) protein in the clinical outcome of IBD.

## GCs

**Figure 1:**
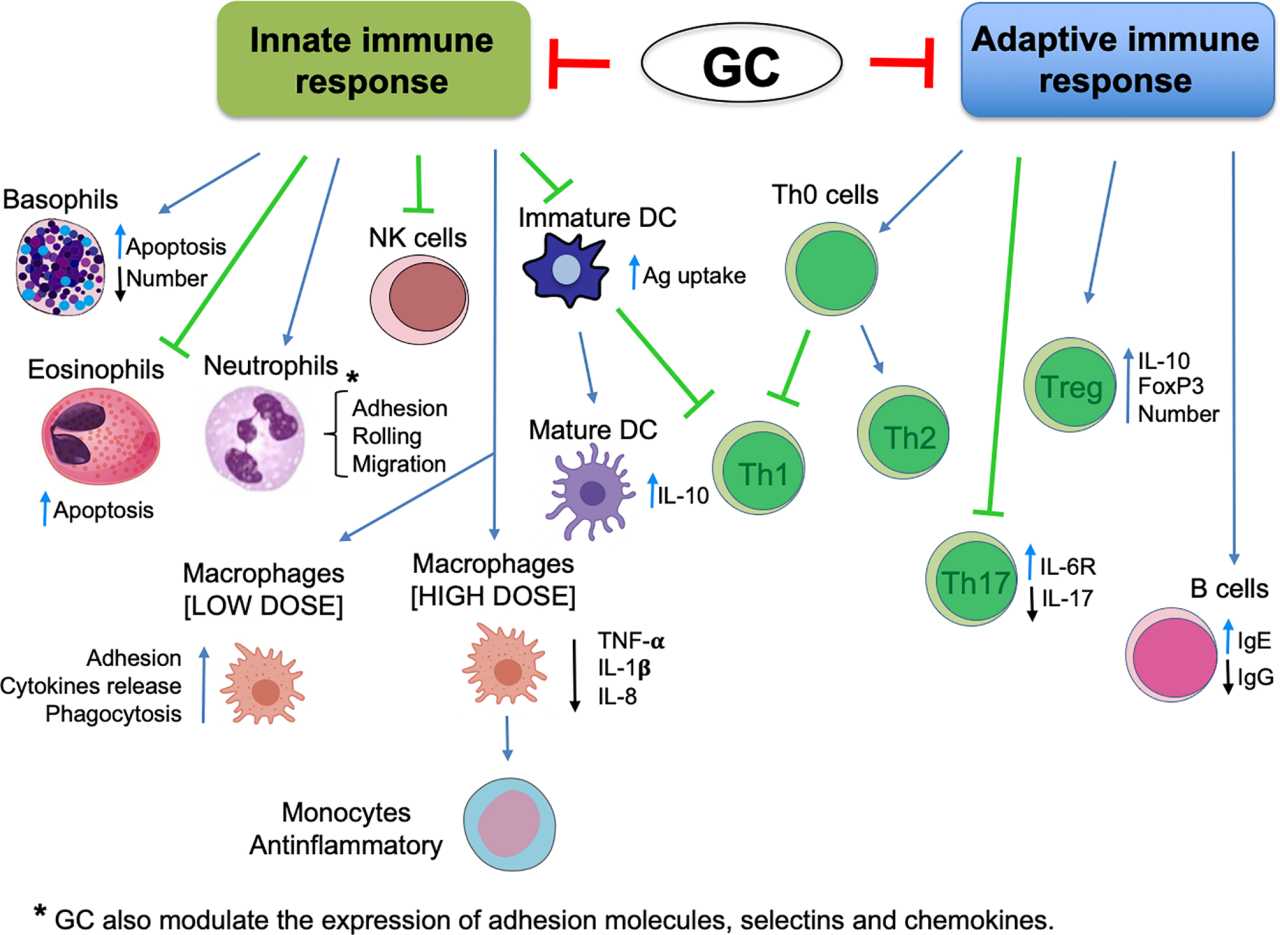
Glucocorticoid effects in innate and adaptive immune response (schematic).

GCs are endogenous molecules (cortisol, cortisone, corticosterone) that can also be synthesized in the laboratory and widely used as treatments for several diseases. GC secretion is mediated by the hypothalamic–pituitary–adrenal (HPA) axis and is regulated by the circadian rhythm and stress, but also by inflammatory stimuli. For example, upon activation by cytokines such as interleukin (IL)-1, tumor necrosis factor, (TNF) and IL-6 (which can lead to extreme tissue damage due to uncontrollable inflammation), the HPA triggers the production and secretion of GCs. The HPA acts on inflammation by regulating genomic and non-genomic pathways, thereby blocking the activation, migration, and proliferation of immune cells of the innate and adaptive immune system (**Figure 1**) (4). Their action is mediated mainly by the GR, which is present predominantly in the cell cytoplasm, bound to a multi-protein complex of heat-shock proteins, immunophilins, kinases, and phospholipases (receptosome). After GC/GR interaction and the consequent conformational changes, the GR dissociates from the receptosome and translocates to the nucleus. The genomic action of the activated GR is elicited by a DNA-binding sequence with two zinc finger motifs. This sequence targets specific glucocorticoid-responsive elements (GREs) to control expression of many genes, such as proinflammatory mediators and transcription factors [e.g., activator protein-1, nuclear factor-kappa B (NF-κB)] (5, 6). Conversely, GC/GR upregulate expression of lipocortin-1, IL-1 receptor antagonists, and Iκ-B. These transcriptional effects contribute to the anti-inflammatory and immunoregulatory actions of GCs. An important target of GC/GR transcriptional activity is GILZ, which interferes with the mitogen-activated protein kinase (MAPK) pathway and NF-kB transcriptional activity and, consequently, regulates inflammatory and immune-mediated reactions (7–10).

Nonetheless, GRE presence is not the only mechanism by which the GR regulates its transcriptional activity. The cell type-specific activities of GCs are dependent upon several other factors, including the chromatin accessibility of target genes, which modulates the availability of DNA sequences (11). Non-genomic actions also contribute to the complexity of GC/GR signaling. These are characterized mainly by a short delay of action and often involve generation of intracellular second messengers and various signal-transduction cascades, such as phospholipase activation, modulation of cyclic adenosine monophosphate, protein kinase pathways, and Ca^2+^ mobilization (12, 13).

GCs have an essential role in controlling the metabolism of carbohydrates, lipids, proteins, water, Ca^2+^, and other electrolytes, as well as maintaining the optimal function of the endocrine, musculoskeletal, cardiovascular, nervous, and immune systems (14–16). Prolonged therapy with GCs amplifies the physiological effects of endogenous GCs, which often lead to deleterious side effects such as hypertension, hyperglycemia, osteoporosis, Cushing’s syndrome, and mood disorders (17, 18). Therefore, the anti-inflammatory and immunosuppressive clinical effects of GCs, even if they are considerable, are transitory. The influence of GCs on the complex control of cellular responses to stress and in the regulation of inflammatory processes is linked markedly also to the adverse effects that accompany their chronic use, and therapy must be discontinued.

## IBD

IBD represents a hypernym for the chronic remission and relapse of an immunologically mediated chronic and lifelong disease characterized by gastrointestinal-tract inflammation. IBD is caused by the interaction between the genetic predisposition of the individual and activation of the immune response. Various environmental factors also have important roles in IBD onset. UC and CD are chronic and debilitating diseases that are incurable (19, 20). T-helper type 2 (Th2) cells have been demonstrated to have a pathogenic role. An imbalance in Th17/T regulatory cells has been linked to UC. CD is characterized by an imbalance in the number of Th1 cells (21). However, recent researches reported a number of novel immune cell populations that correlate with disease in IBD patients indicating that there is no clean cut that UC and CD could be separated according to Th1 and Th2 action (22). The role of B cells is less clear because they mainly control mucosal homeostasis, antibody production, and co-stimulation of T lymphocytes. Experimental evidence suggests that cytokine production by B cells may also affect immune regulation (23, 24).

UC and CD show heterogeneity in terms of clinical and pathological features, and can be distinguished by their location and the nature of inflammation they cause. UC attacks mainly the colonic mucosa, whereas CD can affect the gastrointestinal tract at any level. UC causes inflammation and ulceration of the inner lining of the colon and rectum, and usually manifests with diarrhea, loss of appetite/weight, abdominal pain, fatigue, and anemia. The level of inflammation determines disease severity and the subsequent therapeutic strategy. CD is a chronic inflammatory disorder that can affect all parts of the gastrointestinal tract, but mainly influences the terminal ileum, caecum, perianal area, and colon. Several symptoms are caused by CD, which hampers confirmation of the diagnosis. Among those symptoms are weight loss, bowel obstruction, abdominal pain, fever, and chronic or nocturnal diarrhea, although less frequently than other symptoms. These are all critical parameters for the initial diagnosis (25, 26).

IBD at any age is considered a risk factor for the onset of colon cancer due to the development of chronic inflammation that causes immune-mediated tissue destruction. The disease manifests itself in genetically susceptible individuals (i.e. mutations in IBD susceptibility loci including those of STAT3, TYK2, JAK2, CARD9 and IL10RA genes) (27), as a cause or consequence to an imbalance in commensal microbes, or if different environmental factors favor dysbiosis; poor hygiene (28), unbalanced diets (29), smoking, and stress (27). Several factors have been found to impair the functions of immune cells, including lymphocyte activation, autophagy, GC response, and chemotaxis (2, 30).

Specific and curative treatment for IBD is lacking. The focus is on supportive measures as well as the use of anti-inflammatory and immunosuppressive drugs (e.g., aminosalicylates, GCs, anti-TNF agents, such as infliximab, adalimumab, certolizumab pegol and CDP571) (31). The goals of therapy are to improve the quality of life, reduce the risk of disease-related complications, and avoid the need for surgery.

## First-Generation GCs

**Figure 2:**
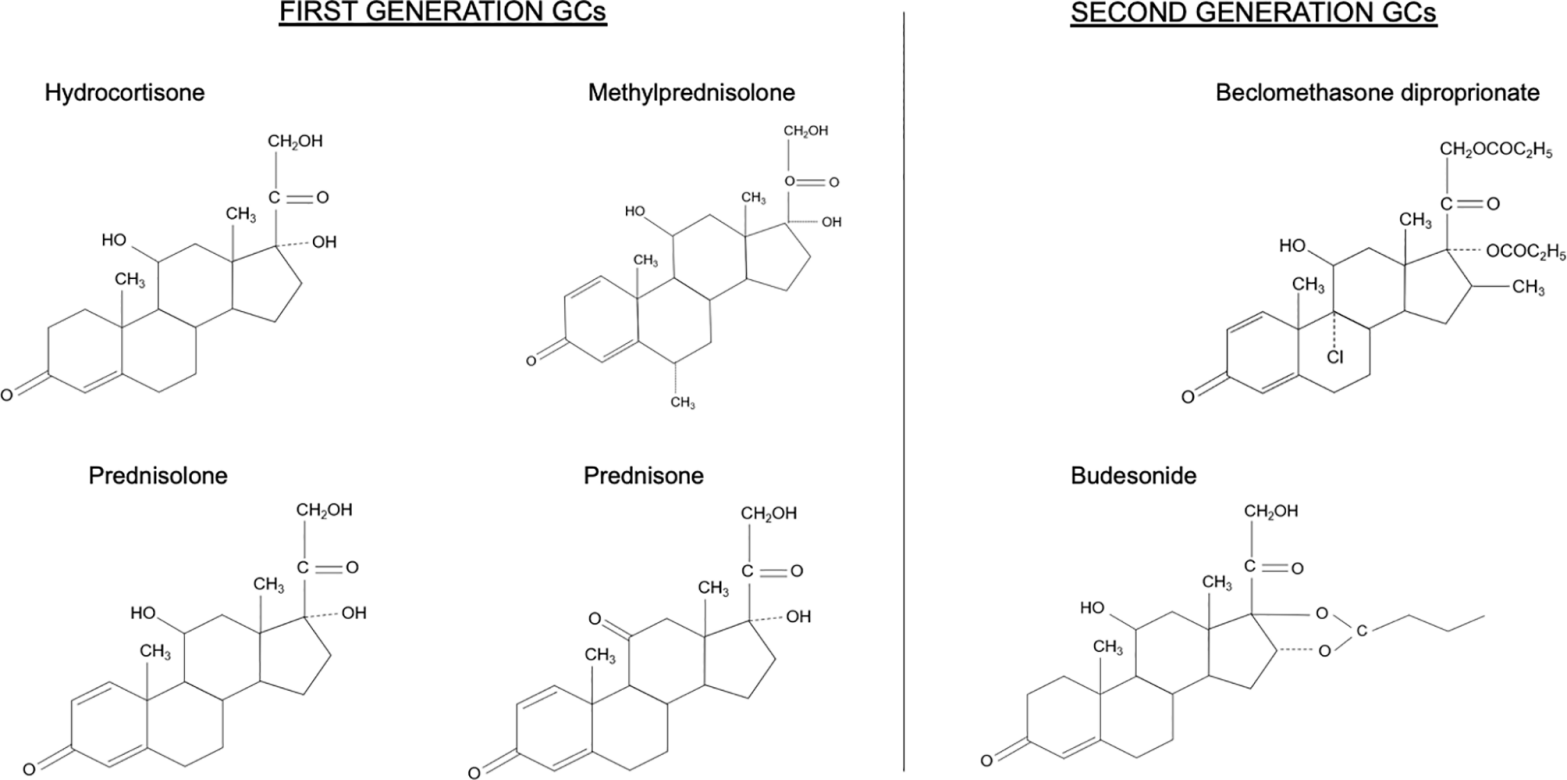
Chemical structures of first and second generation glucocorticoids (GCs).

All GCs can be considered to be valid options when treating IBD. The most commonly used GCs are prednisone, methylprednisolone, and hydrocortisone (**Figure 2**). They can be used alone or in combination with mesalamine for the induction and maintenance of clinical remission in patients suffering from chronic IBD (32–34).

The primary purpose of GC use is to induce clinical remission. A controlled therapeutic regimen guarantees a reduction in the symptoms and side effects caused by these drugs. The most efficacious dose of prednisone is 1 mg/kg up to 40–60 mg/day, and a higher dose seems to not change therapy efficacy (35). The dose administered initially is 40–60 mg/day, but it must be reduced gradually to achieve therapy interruption while maintaining control of IBD symptoms. At the same time it has been shown that alternate-day treatment helps to reduce and prevent the adverse effects on the central nervous system. Concomitant use with other drugs (e.g., azathioprine) also permits to avoid further use of GCs, while allowing maintenance therapy (36). It is important that prednisone can be considered lifesaving in severe diseases and this is especially true of chronic cases of UC after treatments from six weeks to three months. The effects are however temporary, as patients tends to miss out on the benefits after nine months of therapy (37).

It has been shown that intake of first-generation GCs, such as methylprednisolone (12–48 mg/day), if combined with other drugs, such as sulfasalazine (3 g/day), is important for symptom improvement. It was shown that these improvements occurred faster than if patients were treated with sulfasalazine alone (3).

Another factor in the choice of treatment (and consequently the dose) is the administration route because this is dependent upon the disease location. In people suffering from UC, drug administration can be topical if proctitis is present, or topical and systemic in the case of left-side colitis. In CD, disease severity is the main criterion for GC use because patients might not respond well to treatment and might need surgery if disease severity is high (38). To note, non-response to GCs is comparable between CD and UC.

Despite the efficacy of first-generation GCs, several serious side effects can be associated with their use, including osteoporosis, hyperglycemia, hypertension, mood disorders, gastric ulcer, and increased susceptibility to infections (3, 17). This characteristic limits their long-term use. Maintenance treatment with systemic GCs is not recommended.

## Second-Generation GCs

Due to the limitations of prolonged therapy with first-generation GCs, compounds such as budesonide and BDP have been developed that maintain topical anti-inflammatory efficacy but minimize their bioavailability and, consequently, their systemic adverse effects (**Figure 2**) (3).

Budesonide undergoes hepatic inactivation before reaching the systemic circulation to diminish corticosteroid-related side effects. Several clinical trials have been conducted to establish the efficacy of oral budesonide. A randomized, double-blind controlled trial conducted in patients with mild, active CD demonstrated that budesonide, if administered at 9 mg/day for 2 months, was as efficacious as prednisone (which was used at 40 mg/day for the first 2 weeks) followed by 30 mg/day in the third month (39). Budesonide carried a lower prevalence of systemic adverse effects (33% of cases) than that of conventional GCs (55%). Budesonide use has not been reported to directly cause changes in the bone mineral density or adrenal insufficiency, however individual sensitivity may vary and therefore cause their onset. Consequently there is no direct correlation to the appearance of osteoporosis symptoms or decreased/inadequate cortisol production (40, 41). Multiple meta-analyses have demonstrated the efficacy of budesonide in inducing remission, and that a once-daily dose is just as efficacious as 3 mg taken thrice daily. In the context of more severe disease, budesonide is not as efficacious as prednisolone in achieving remission (42).

The second-generation GC BDP is a derivative of cortisone. It has topical effects and minimal systemic activity. It is administered as a prodrug and is partially metabolized in the lower gastrointestinal tract. If used at low doses, BDP has been shown to be free of many of the deleterious side effects associated with GCs absorbed systemically. The induction of remission in CD using BDP has not been investigated in double-blind randomized trials. The efficacy of BDP in patients with UC has been well established: eight double-blind randomized trials have been published (43). Oral BDP for UC has been approved in several countries. In one randomized controlled trial, oral BDP was administered as a daily dose of 5 mg for 4 weeks, then alternated weekly for 4 weeks. A similar efficacy to that observed with prednisolone was evident, and there was no difference in the prevalence of adverse events. However, as a second-generation GC with low systemic absorption, it is associated with fewer adverse events than that of first-generation GCs (44, 45).

## New Therapeutic GC-Based Approaches in Chronic IBD

Several studies have aimed to develop new delivery methods for existing drugs to improve the efficacy of therapeutic approaches by limiting the side effects associated with the use of specific drugs or resistance to therapy. Complexity of molecular pathways and networks regulating living organisms has become a central issue of molecular biology. Thus, “molecular stratification” is a new approach based on the needs of patients, their susceptibility to disease, and their gene variability. Studies are carried out on specific elements belonging to different molecular pathways. This can take the form of choosing biologically active molecules bound to cells such as erythrocytes, the targeting of selective GR agonists or the targeting of specific elements able to regulate the inflammatory reaction upon their activation. *GILZ* is a target linked to GC action, so it can be considered a new approach for the regulation of inflammatory diseases such as IBD or even as a molecular marker of inflammation (10, 24).

### Drug-Delivery Strategies in IBD Therapy

Drug-delivery systems aim to improve the pharmacological activities of drugs. They play a key part in regulating solubility, drug aggregation, low bioavailability, poor biodistribution, and reducing side effects (46).

Red blood cells (RBCs) are the largest population of blood cells. Their main function is to carry oxygen to all body tissues. To use erythrocytes as carriers of biologically active substances, pores must be formed reversibly in their membrane, through which the drug can penetrate. To achieve this aim, the cell is stimulated with external influences, such as ultrasound. Drug molecules can also enter by endocytosis in the presence of certain chemical compounds, such as hydrocortisone. Active substances of different molecular weights can be delivered by incorporation in the cell or by binding to its surface (47). The average life of RBCs guarantees a therapeutic effect for 120 days, so biologically inactive compounds such as dexamethasone-21-phosphate are used (48). In recent years, drug selection and the relationship between specific drugs and RBCs have been adapted to reduce some of the limitations that their use could present. Prodrugs, such as corticosteroid prodrugs and nucleotide prodrugs, can improve the action of RBCs as carriers. If the molecule to be delivered is inactivated, erythrocytes can transform and release it in its active form (49).

A type of colonic-delivery technology called Multi-Matrix System (MMX^®^) has been developed to provide controlled release of budesonide throughout the entire colon. The most common adverse effects in studies using MMX were headache, nausea, and urinary-tract infection (50).

Therapy with systemically active GCs, including budesonide MMX, is associated with suppression of endogenous cortisol levels and HPA function. Oral budesonide MMX at 9 mg/day has been shown to be significantly more efficacious than placebo and can induce remission in mild-to-moderate UC, being as efficacious as 5-aminosalicylic acid (5-ASA, which is first-line therapy for mild-to-moderate active UC) (51). GC effects appear in a shorter time, while 5-ASA takes approximately 2 weeks and therefore it cannot be used in moderate disease as a sole therapeutic regimen (52).

Another strategy for drug delivery involves nanoparticles (NPs) which are organic, inorganic or polymeric particles that ranges between 1 to 100 nanometres in size. Among synthetic polymers used in pharmaceutical formulations there are poly-L-lactic acid, polyvinyl alcohol, poly(lactic-co-glycolic acid), and polyethylene glycol (53). NP efficacy is linked mainly to rapid and preferential intestinal uptake and decreased excretion of the drug following diarrhea (one of the main symptoms of IBD). Cellular internalization of NPs is characterized by paracellular transport or endocytosis into epithelial cells. It has been shown that NPs of diameter 200 nm prepared with cetyltrimethylammonium bromide and relatively neutral charge adhered to healthy colonic tissue in controls as well as inflamed colonic tissue in a colitis model using 2,4,6-trinitrobenzene sulfonic acid (TNBS). Simultaneously, polylactide–coglycolide FK506-NPs were shown to enhance drug penetration into inflamed tissue significantly in a TNBS-induced model and oxazolone-induced colitis model. Other parameters capable of influencing uptake (in addition to diameter) must be studied in detail. *In vivo* and *ex vivo* studies have shown that the surface charge of NPs can affect targeting in the colon. Cationic systems adhere to the mucosal surface due to the interaction between the positively charged nanocarrier and the negatively charged intestinal mucosa. Anionic systems adhere preferentially to targeted sites *via* electrostatic interaction with the higher concentration of positively charged proteins (53). Use of hydrophilic and uncharged NPs is also under development. The creation of NPs bound to the surface with poly(ethylene glycol) (PEG) seem to increase NP translocation through the mucus and to ensure a substantially increased half-life. Another type of nanoformulation used for GC delivery is polymeric micelles in which PEG is present to allow an extended circulation time and slower release of the compound (54). Zeng et al. showed that a resin microcapsule composed of 717 anion exchange resin and Eudragit S100 could target dexamethasone to the colon, improve the efficacy of UC therapy, and reduce the overall toxic side effects (55).

### Selective Glucocorticoid Receptor Agonists (SEGRAs)

Identification and development of SEGRAs is of particular interest because their therapeutic potential is uncertain (56, 57). SEGRAs are also defined as “dissociated GCs” because they could allow anti-inflammatory activities and the avoidance of metabolic effects. SEGRAs allow the mediation of only repressive effects on various controlled target genes from the GR, and not those of gene activation, because they work by operating through one of two main pathways: transactivation or transrepression (58). The concept that the activating action is responsible for the metabolic (and therefore collateral) effects, whereas immunosuppressive mechanisms are dependent upon the repressive effects, is controversial. Some SEGRA compounds have been shown to have anti-inflammatory properties with reduced collateral effects, such as Compound A [2-((4-acetophenyl)-2-chloro-N-methyl) ethylammonium-chloride))], or ZK216348 N-(4-methyl-1-oxo-1H-2,3-benzoxazine-6-yl)-4-(2,3-dihydrobenzofuran-7-yl)-2-hydroxy-2-(trifluoromethyl)-4-methylpentanamide (59). Therefore, their efficacy *in vivo* for treatment of inflammatory diseases, such as colitis, asthma, and rheumatoid arthritis, is being evaluated (60, 61).

### GILZ-Based Therapy

**Figure 3:**
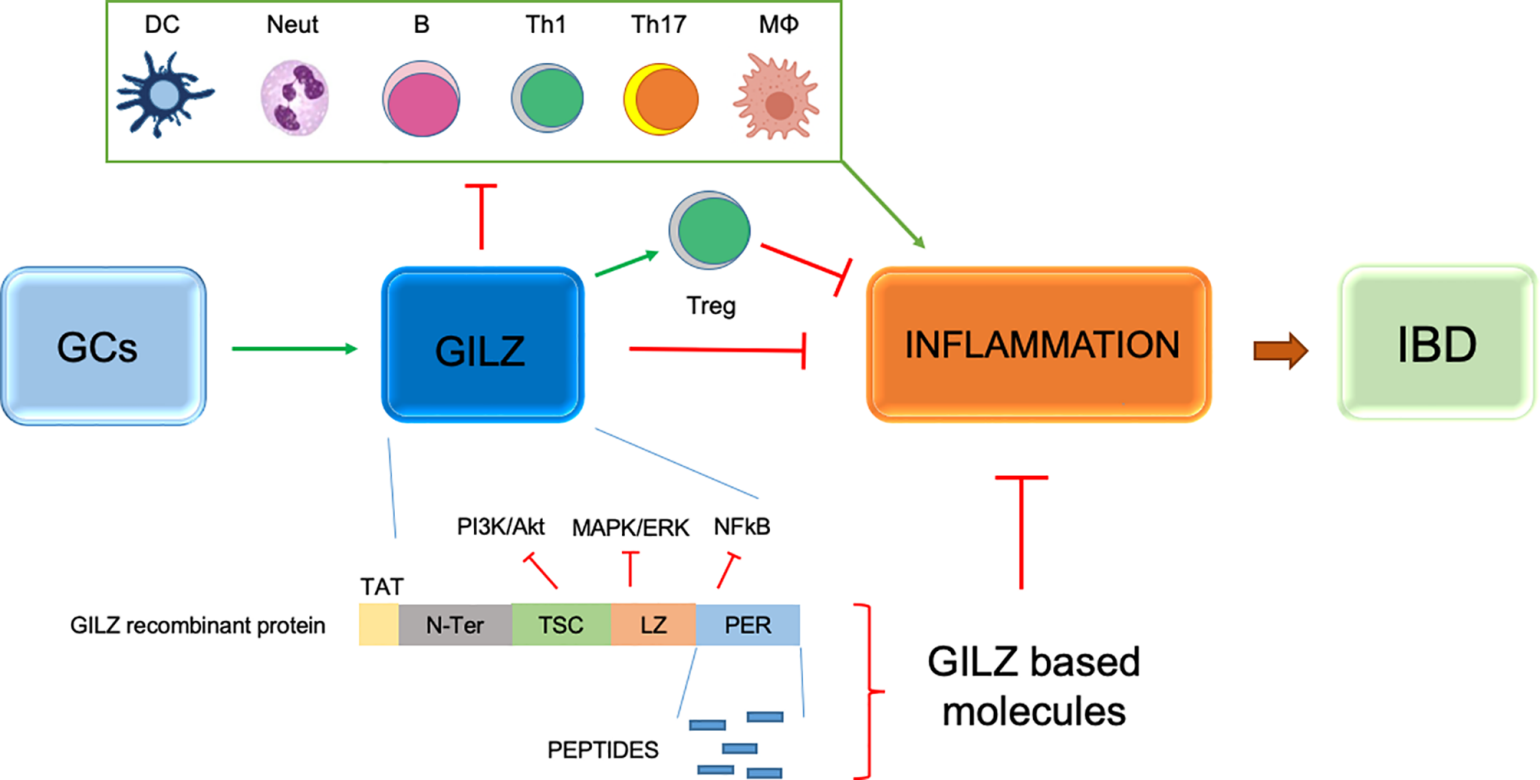
Schematic representation of GILZ efficacy in inflammatory processes and IBD and anti-inflammatory activity of GILZ based molecules, such as peptides deriving from the Proline anf Glutamic acid rich (PER) region of the GILZ protein and TAT-GILZ full length protein. Green arrows indicate positive regulation; red lines indicate negative regulation. DC, dendritic cells; Neut, neutrophils; B, B cells; TH1, T helper Type-1 cells; Th17, T helper Type-17 cells; MФ, monocytes/macrophages; T regulatory cells.

One possible therapeutic solution involves GILZ being induced rapidly by CGs. GILZ protein has been recognized as a downstream inducer of anti-inflammatory effects (**Figure 3**). GILZ is a protein of 135 amino acids. It is highly conserved in humans and mice, and its binding activity can interfere with MAPK/extracellular receptor kinase and protein kinase B pathways (62, 63). Its action is mediated by the binding of GRs to the GREs present in the proximal region of the GILZ transcriptional start site. At the molecular level, GILZ can interact with proteins such as NF-κB, thereby obstructing the transcription of proinflammatory mediators (64). GILZ is expressed in virtually all cells, including macrophages, granulocytes, dendritic cells and T lymphocytes, as well as other non-lymphoid tissues (10). The function of GILZ has been identified only partially and its role (like that of GCs) is being studied in several mouse models of inflammatory and autoimmune diseases. Attention has focused mostly on various transduction pathways that act on suppression of the immune system (65). Several studies have focused on the role of GILZ and analyzed its protective aspect in dinitrobenzene sulfonic acid (DNBS)-induced colitis. It has been shown that transgenic mice overexpressing GILZ had lower levels of colonic inflammation. This result was reliant on a decrease in the number of Th1 cells in the lamina-propria lymphocytes and increase in the number of Th2 cells induced by GILZ overexpression (24, 66). Also, treatment with a recombinant form of TAT-GILZ has been studied in animals with IL-10 knockout. The presence of GILZ has been shown to lower the prevalence of spontaneous UC, thereby demonstrating a protective action of GILZ against severe inflammation (66). Moreover, we and other groups demonstrated the efficacy of GILZ based molecules (full recombinant GILZ protein or peptides) in different experimental models of inflammatory diseases. The main inflammatory target of GILZ is NF-kB. GILZ binds and inhibits p65/NF-kB subunit, thus exerting its anti-inflammatory activity (7, 64). The GILZ interaction with NF-kB is mediated by proline-rich (PXXP) domains present in proline and acid glutamic rich (PER) region of GILZ COOH-terminal portion. Consequently, the possibility to synthesize and use GILZ peptides, that include PXXP domains of the GILZ protein, could be an alternative to the administration of the entire recombinant GILZ protein with possible pharmacokinetic and pharmacodynamic advantages. Notably, no apparent toxic effects have been observed in mice upon *in vivo* administration (intraperitoneally or subcutaneously) of TAT-GILZ protein or GILZ-peptides in mice (66, 67, and Bruscoli, unpublished data). Thus, GILZ represents a new approach for IBD treatment, and to have an immunosuppressant effect comparable with that of GCs (24, 66, 68). GILZ has also has been associated with several inflammatory diseases other than IBD, such as rheumatoid arthritis and fibromyalgia (69, 70). An advantage of GILZ therapy could be that side effects have not been associated with administration of GILZ recombinant protein in cellular systems or in established *in vivo* models of IBD.

Therefore, novel drugs in inflammatory/autoimmune diseases (including IBD) can be developed based on the structure and molecular interactions of GILZ. Moreover, GILZ could be of interest for several inflammation-based degenerative diseases and possibly cancer.

## Author Contributions

SB and MF wrote the article. CR substantially contributed to the conception and design of the article and interpreting the relevant literature. GM revised it critically for important intellectual content. All authors contributed to the article and approved the submitted version.

## Funding

This work was supported by the Crohn’s & Colitis Foundation, award number 504854, USA and by the Italian Ministry of Education and Research, grant PRIN 2017-2017XZMBYX.

## Conflict of Interest

The authors declare that the research was conducted in the absence of any commercial or financial relationships that could be construed as a potential conflict of interest.
